# Genetic Evidence for Multiple Sources of the Non-Native Fish *Cichlasoma urophthalmus* (Günther; Mayan Cichlids) in Southern Florida

**DOI:** 10.1371/journal.pone.0104173

**Published:** 2014-09-03

**Authors:** Elizabeth Harrison, Joel C. Trexler, Timothy M. Collins, Ella Vazquez-Domínguez, Ulises Razo-Mendivil, Wilfredo A. Matamoros, Christian Barrientos

**Affiliations:** 1 Department of Biological Sciences, Florida International University, Miami, Florida, United States of America; 2 Departamento de Ecología de la Biodiversidad, Universidad Nacional Autónoma de México, México DF, México; 3 Intituto de Ecologia A. C., Red de Biología Evolutiva, Veracruz, Mexico; 4 Department of Biological Sciences, University of Southern Mississippi, Hattiesburg, Mississippi, United States of America; 5 Department of Fisheries and Aquatic Science, University of Florida, Gainesville, Florida, United States of America; Chinese Academy of Fishery Sciences, China

## Abstract

The number and diversity of source populations may influence the genetic diversity of newly introduced populations and affect the likelihood of their establishment and spread. We used the cytochrome b mitochondrial gene and nuclear microsatellite loci to identify the sources of a successful invader in southern Florida, USA, *Cichlasoma urophthalmus* (Mayan cichlid). Our cytochrome b data supported an introduction from Guatemala, while our microsatellite data suggested movement of Mayan Cichlids from the upper Yucatán Peninsula to Guatemala and introductions from Guatemala and Belize to Florida. The mismatch between mitochondrial and nuclear genomes suggests admixture of a female lineage from Guatemala, where all individuals were fixed for the mitochondrial haplotype found in the introduced population, and a more diverse but also relatively small number of individuals from Belize. The Florida cytochrome b haplotype appears to be absent from Belize (0 out of 136 fish screened from Belize had this haplotype). Genetic structure within the Florida population was minimal, indicating a panmictic population, while Mexican and Central American samples displayed more genetic subdivision. Individuals from the Upper Yucatán Peninsula and the Petén region of Guatemala were more genetically similar to each other than to fish from nearby sites and movement of Mayan Cichlids between these regions occurred thousands of generations ago, suggestive of pre-Columbian human transportation of Mayan Cichlids through this region. Mayan Cichlids present a rare example of cytonuclear disequilibrium and reduced genetic diversity in the introduced population that persists more than 30 years (at least 7–8 generations) after introduction. We suggest that hybridization occurred in ornamental fish farms in Florida and may contribute their establishment in the novel habitat. Hybridization prior to release may contribute to other successful invasions.

## Introduction

Biological invasions have resulted in species declines, extinction of native biota, and extensive financial costs [1], [2]. Some of the largest impacts of nonnative species have been recorded in aquatic habitats [3], [4]. Since European colonization, southern Florida has experienced major habitat transformation and invasion by approximately 1200 nonnative species [Floridainvasives.org]. Florida's highly disturbed landscape and mild subtropical climate foster the establishment of tropical species [2], [5]–[7], including fish [7], [8]. Approximately 196 fish species have become established in Florida [9],mostly through the aquarium trade [8], [10]–[12] which also enhances the probability that introductions from multiple sources occur, especially in a major shipping and transportation hub such as southern Florida [13], [14]). Identifying the route of invasion and the source populations of invaded areas can improve the quality of management strategies for the invader either within the source range, the pathway of invasion or the method and point of entry into the invaded regions [15].

Identification of sources and pathways of invasions has traditionally been accomplished by examining historical data such as dates of first discovery in introduced areas and importation records, or by molecular analyses of native and introduced populations [16]. Historical data alone are not usually enough to infer introduction pathways as they may be incomplete or insufficient to distinguish successful and unsuccessful establishment and spread. Molecular methods facilitate the comparison of genetic diversity of native and introduced populations to narrow the viable hypotheses of origin and spread. However, these methods are limited to *post hoc* assumptions about the genetic effects of introductions and demographic stochasticity; the challenge that unsampled populations might be the true source should also be considered [17]. Approximate Bayesian Computation (ABC) and coalescent theory allows for the statistical comparison of complex introduction pathways that incorporate changes in population size, admixture before or during introduction, and historical and biogeographical data [18], thus alleviating some of the limitations of molecular analysis.

Non-native species are typically assumed to be under strong selective pressure to adapt to their new environment, become established, and spread [13], [19], [20], but introduced populations often have low genetic diversity from founder effects and population bottlenecks that may limit their ability to respond to environmental challenges (the ‘invasive species paradox’ [21]). One resolution of this paradox is that multiple introductions of an invasive species are correlated with successful establishment, especially if the introductions arose from two or more genetically distinct sources [22]–[24]. Introductions from multiple sources may produce novel genetic combinations that increase fitness and facilitate invasion success [24]–[30]. On the other hand, limited introductions and subsequent genetic bottlenecks do not necessarily decrease genetic diversity [31] and establishment can still occur after genetic bottlenecks [32]–[34]. Studies have documented establishment of nonnative species resulting from multiple introductions, or introduction from multiple sources [23], [35], [36], as well as from single introductions or extreme bottlenecks [37], [38]. Establishment can thus be influenced by many factors and each introduction should be examined individually.

Cytonuclear disequilibrium, the nonrandom association of organellar haplotypes and nuclear alleles, has been documented for interspecific hybrids [39]–[43] and in host-parasite interactions [44], [45]. Cytonuclear disequilibrium may result from several demographic phenomena including nuclear-organellar genotypic interactions affecting fitness, genetic drift in small populations, founder effects preceding rapid population expansion, and nonrandom mating from geographically patterned admixture, migration, and hybridization (summarized and discussed in [46]–[48]). A nonrandom relationship between organellar and nuclear genes is expected as a result of species introductions from multiple sites, which are accompanied by population bottlenecks and admixture of distinct genomes [46], [49], [50].

At least 13 species of cichlids have become established in Florida, which possesses no native members of the family Cichlidae [51]. *Cichlasoma urophthalmus* (Mayan Cichlid) is found in freshwater and salt water along the Atlantic slope of Central America including southern Mexico, Belize, Guatemala, Honduras and Nicaragua [52]. Mayan Cichlids are economically important to artisanal fisheries and aquaculture in their native range [53], [54]. They were first recorded in southern Florida in the Everglades National Park in 1983 [55]. Since then, Mayan Cichlids have spread over approximately 70,000 hectares from southern to central Florida during the 30 years since they were introduced (at least 7 generations [56]–[59]). Mayan Cichlids have successfully established in the southern Florida environment across a range of salinities from freshwater marshes to 40 psu in the mangrove zone, where they can dominate the fish communities [59], [60]. They have been shown to alter the relative abundance of native fish populations, most likely by predation [60]–[62].

Successful establishment of a nonnative species depends on many factors and varies with species. Understanding the origin and method of introduction of nonnative species is necessary for developing effective ecosystem management strategies and for preventing future introductions. A reconstruction of invasion pathways is needed to understand the effects of diversity of introductions, the number of founder individuals, and the combination of historically separate genotypes on introduced populations. We used mitochondrial and nuclear molecular markers to identify the source(s) of Mayan Cichlids in Florida to determine whether this successful invader resulted from single or multiple introductions.

## Materials and Methods

### Ethics Statement

This study was carried out in strict accordance with the recommendations in the Guidelines for The Use of Fishes in Research of The American Fisheries Society, the American Institute of Fisheries Research Biologists, and the American Society of Ichthyologists and Herpetologists [63]. The protocol was approved by the Institutional Animal Care and Use Committee of Florida International University (Protocol approval number 08-014). Fin clippings were obtained from some fish by nonlethal means. Some fish were euthanized in a solution of 0.02% MS-222 (Tricaine methanesulfonate) and preserved for collections at Florida International University. All efforts were made to minimize suffering. Our study did not involve endangered or protected species. Samples from Chichén-Itza, Mexico, were collected under a permit issued by Instituto Nacional de Antropología e Historia; specific permission was not required for collection from other regions in Mexico. Samples from Honduras were collected under a permit issued by Instituto de Conservacion Forestal (ICF); samples from Nicaragua were collected under a permit issued by Miniserio del Ambiente y los Recursos Naturales (MARENA); samples from Guatemala were collected under a permit issued by National Council for Protected Areas; samples from Belize were collected under a permit issued by the Belize Ministry of Agriculture and Fisheries. Samples collected in Florida were collected under a permit issued by Florida Fish and Wildlife Conservation Commission.

### Sample Collection

**Figure 1:**
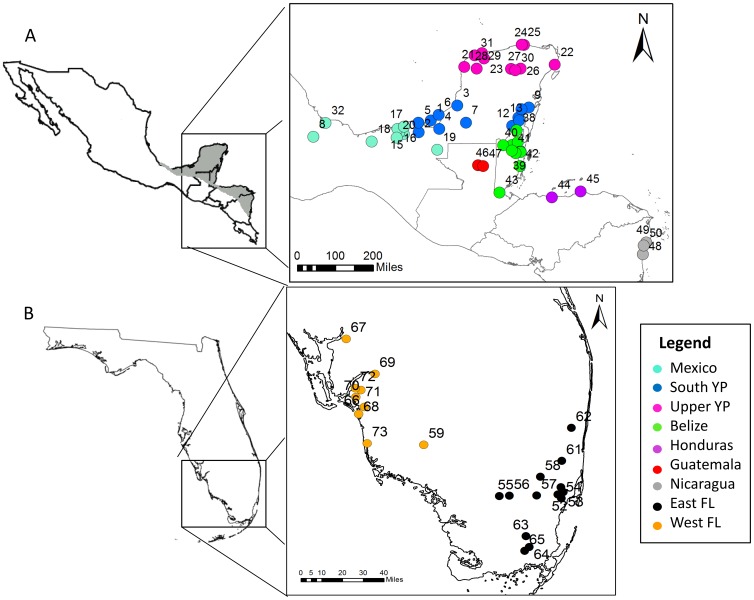
Map of sampling sites for Mayan Cichlids in Mexico and Central America (A) and Florida (B). Numbers on the map correspond to site numbers in Table S1. Light grey shading in box A indicates the range of Mayan Cichlids (Miller 1966) in the native range. “Mexico” denotes samples from Mexico that are not within the Yucatán Peninsula (states of Yucatán, Campeche and Quintana Roo). YP = Yucatán Peninsula; FL = Florida.

We collected tissue samples from 670 individual Mayan Cichlids from 23 sites in Florida (287 individuals) and 53 sites within Mexico and Central America (383 individuals), including sites in Belize, Honduras, Guatemala and Nicaragua (Table S1; Figure 1). Fish were captured using a combination of methods: hook-and-line, cast net, throw trap, seine and minnow trap in habitats that ranged from freshwater ponds to estuarine canals and mangrove habitats. In some regions of Mexico and Belize, fish were purchased from local fishermen as they were coming to shore. Some fin clippings were also obtained from sample collections at the Universidad Nacional Autónoma de México (UNAM). We also acquired two specimens from a pet store in North Miami, Florida, USA, which had obtained them from a local fish farm, and included these specimens in mitochondrial analyses. Samples were either frozen or fixed in 90% ethanol. Total genomic DNA was isolated from either muscle or fin tissue using the DNeasy Blood and Tissue Kit (Qiagen) following the manufacturer's protocol.

### Molecular Analyses

#### Mitochondrial gene

A portion of the cytochrome b mitochondrial gene was amplified using CytbFor5′-TGATGAAACTTCGGCTCCC-3′ and CytbRev5′-CTGTTAGTCCGGCGATAGG-3′. These primers were designed specifically for this study using primers designed by [64]. The PCR reactions were carried out in a 50 µL volume using 10 µL of 5× reaction buffer, 3 µL of 25 mM magnesium chloride, 2.5 µL each of 10 mM forward and reverse primers, 1 µL of 10 mM dNTP's, 0.5 µL of Taq DNA Polymerase (5 µ/µL), 2 µL of the DNA sample (approximately 10–200 ng) and 28.5 µL of Sigma sterilized water. Amplifications were conducted for cytochrome b with a MJ Research thermal cycler using standard methods. Thermal cycling conditions for cytochrome b consisted of an initial hot start of 55°C (10 min), then 36 cycles of 95°C (30 seconds), 55°C (45 seconds, 72°C (45 seconds), followed by 49°C (1 minute). A final incubation of 72°C for 4 minutes was added to ensure complete extension of amplified products. Subsequently, PCR products were subjected to gel electrophoresis in a 1.4% agarose gel run in Tris-Borate-EDTA (TBE) buffer followed by staining with ethidium bromide and visualization with UV light. For sequencing, positively amplified DNA was then purified using 2 µL of ExoSap per 5 µL of PCR product. Samples were then sequenced using Big Dye Terminator version 3.1 on a 3130XL Genetic Analyzer (Applied Biosystems). For sequencing, the internal primers designed were: CytbIntF5′-CACCAACCTCCTCTCCGC-3′ and CytbIntR5′-TGGAAGGCAAAGAATCGGG-3′.

Initially, 47 fish from four sites in Florida, four sites in Mexico, two sites in Belize and one site in Honduras were sequenced for a portion of the cytochrome b gene (851 bp). These sequences revealed six haplotypes, two of which were found in 43 individuals. The two haplotypes differentiated between fish from Mexico and Central America and fish from Florida, hereafter referred to as the CA haplotype and the Fl haplotype respectively; on the basis of those results, we screened the remaining samples for those two haplotypes using restriction endonucleases. Cytochrome b was first amplified using Polymerase Chain Reaction (PCR). Positively amplified DNA was then digested with EcoRV at 37°C for one hour. EcoRV digestion resulted in two fragments if an individual displayed the Fl haplotype and one fragment if the CA haplotype was present. DNA fragments were then separated electrophoretically, stained with ethidium bromide and viewed under UV light. The remaining 623 samples were screened for the CA and Fl haplotypes.

#### Nuclear markers

Specimens from 357 individuals from 29 sites in Florida, Mexico, Belize, Guatemala, Honduras and Nicaragua were analyzed using 17 recently developed microsatellite nuclear markers (see [65] for primer information). We amplified DNA from fish for sites where we had collected at least 10 specimens. The PCR reactions were carried out in 10 µL using 1 µL of 5× reaction buffer, 1 µL of 25 mM magnesium chloride, 0.5 µL each of 10 mM forward and reverse primers, 0.2 µL of 10 mM dNTP's, 0.2 µL of Taq DNA Polymerase (5 µ/µL), 1 µL of DNA sample (approximately 10–200 ng) and 5.6 µL of Sigma sterilized water. Touchdown PCR cycling parameters were run on an MJ Research thermal cycler; see [55] for complete protocol. Thermal cycling conditions consisted of: 95°C (5 minutes), then 20 cycles of 95°C (30 seconds), a temperature of 58°C, 60°C, 66°C or 67°C depending on the locus that decreased by 0.5°C per cycle (30 seconds), and 72°C (30 seconds), followed by 20 cycles of: 95°C (30 seconds) 48°C, 50°C, 56°C or 57°C depending on the locus (30 seconds), 72°C (30 seconds), then 72°C for 5 minutes. The PCR products were run on 1.4% agarose gel and prepared for GeneScan using 9.75 µL of Hi Di formamide solution (Applied Biosystems), 0.25 µL of GeneScan LIZ-500 size standard (Applied Biosystems) and 1 µL of PCR product. The PCR products were run on a 3130XL Genetic Analyzer (Applied Biosystems) to determine DNA sizes (DNA Core Facility, Florida International University). Peak Scanner 2 (Applied Biosystems) was used to determine fragment sizes of alleles.

### Data Analyses

#### Mitochondrial data

Sequences were aligned using Sequencer v.4.8 and checked manually. Cytochrome b haplotypes were analyzed using MRMODELTEST 2.3 [66] and MRBAYES 3.2. [67]. We conducted hierarchical hypothesis tests to select the appropriate evolutionary model for subsequent Bayesian phylogenetic analysis. The program MRMODELTEST calculated base frequencies, which were used to model the prior probability distribution; likelihood ratio tests selected the TrN model (equal transversion rates but two different transition rates) for the Bayesian analysis. Bayesian phylogenetic analysis was run for 1,000,000 generations, sampling every 100 generations. We discarded the initial 10% of trees during the ‘burn-in period’ and made a 50% majority consensus rule from the remaining Bayesian trees. The analysis was repeated twice to avoid searching within local optima. The phylogenetic tree was used to identify distinct clades where haplotypes were shared among Mayan Cichlids from southern Florida and from the native range. Unlike typical phylogenetic trees that include taxa on their branches, we replaced the taxa with sampling locations to examine the phylogenetic relationships among sites resulting in a general area cladogram [68].

To investigate the relationships between clades, haplotype networks were built using Network v. 4.6.11 and Network Publisher (http://www.fluxus-engineering.com/). The maximal pairwise difference between sequences was 6 and the tranversion:transition ratio was weighted as 2∶1; we therefore specified the weighted genetic distance (epsilon) as 120 and conducted a median-joining analysis [69] using the greedy distance calculation method [70].

#### Nuclear data

The number of different alleles, the number of effective alleles, observed and expected heterozygosities, inbreeding coefficient (FIS) and percentages of polymorphic loci were calculated for Florida, Upper Yucatán Peninsula, South of Yucatán Peninsula, Belize, Guatemala, Honduras, and Nicaragua using GenAlEx v.6.5 [71], [72].

To detect evidence of a recent bottleneck or reduction in population size of Mayan Cichlids in Florida, we used the software Bottleneck v.1.2.02 [73]. We performed the Wilcoxon signed rank test to test for heterozygosity excess. When a bottleneck occurs, it is expected that both allele frequencies and heterozygosities decrease, however, allele frequency is expected to decrease faster than heterozygosity. Thus, the program Bottleneck tests for heterozygosity excess by comparing expected heterozygosity under Hardy-Weinberg equilibrium to heterozygosity expected under mutation-drift equilibrium determined by the number of alleles [74]. We tested for heterozygosity excess under the Stepwise Mutation Model.

Genetic relatedness of populations was assessed using Bayesian clustering in STRUCTURE v.2.3.4 [75]. STRUCTURE was used to estimate the number of populations (K) most likely present in the samples. The parameters were set using an admixture model with independent allele frequencies and sampling locations were used as priors; values for the level of admixture (alpha) were inferred from the dataset. STRUCTURE analyses were performed using the freely available Bioportal server (http://www.bioportal.uio.no) [76]. The burn-in length was set to 50,000 and the simulation to 500,000 repetitions. Each run was iterated 20 times. We evaluated results for K = 1 to K = 35. To determine the most probable clustering of the data, K was selected using the ΔK approach [77] as implemented by Structure Harvester [78]. The variable ΔK is calculated from the rate of change of the log likelihood of the data between runs with successive values of K [77]. CLUMPP v.1.1.2 [79] was used to summarize parameters across 20 iterations and the corresponding graphical output was visualized using DISTRUCT v. 1. 1 [80].

ABC was used to test different introduction pathways of Mayan Cichlids into Florida using the microsatellite data and the program DIYABC [81]. ABC uses summary genetic statistics (such as genetic distance and the number of alleles) to compare observed and simulated datasets given hypothesized scenarios. Posterior distributions of parameters for the proposed models – possible introduction pathways in our case – are calculated from the differences between the observed and simulated datasets [82], [83]. Hypotheses and scenarios were generated on the basis of the results of phylogenetic analyses of cytochrome b, population assignment by cluster analysis, as well as on historical biogeography and hydrology of the native range (see Table S2 for proposed scenarios). Cytochrome b phylogeny indicated that samples from Belize, Honduras and Nicaragua were within the same clade and cluster analysis also grouped samples from those regions (see Results), although there appeared to be some overlap among individuals from Belize and Florida. Cytochrome b data also showed that samples from both the eastern and western coasts of Florida were within the same clade and also part of the same cluster (see Results).

We tested two groups of scenarios using the software DIYABC v. 2.0 [81] wherein the scenarios increased in complexity by changing the grouping of samples into populations to improve model fit (Table S2). The results from the first group of scenarios informed the second group. The first group contained 15 scenarios that used five distinct populations from Florida, Mexico, Guatemala, a possible unsampled source population, and a grouping of Belize, Honduras and Nicaraguan sites (hereafter referred to as BHN); Belize, Honduras and Nicaragua were grouped together because they shared the same cytochrome b haplotype and were assigned to the same population by Bayesian cluster analysis (Table S2). Samples from East and West Florida were combined into one population because both phylogenetic analysis and cluster analysis grouped them together. In the first grouping of scenarios, we tested whether Mayan Cichlids were introduced into Florida from BHN, Mexico, Guatemala, from both Mexico and Guatemala, or from an unsampled population in Central America. We also included a possible unsampled, ‘ghost’ population of Mayan Cichlids in Central America which, in some scenarios, was the source for populations in Mexico and Guatemala. The second group contained nine scenarios that merged cytochrome b results and hydrology of the region; we separated the Mexican samples into two populations, Upper Yucatán Peninsula (YP) and south of the Yucatán Peninsula, and categorized Belizean samples as a distinct group because the Belizean sites are within the Usumacinta Province [84] unlike the Honduras and Nicaraguan sites, which were grouped together (Table S2). The cenote-rich Upper Yucatán Peninsula lacks any major rivers or drainages that connect it to the regions south of the Peninsula [84], [85], so we treated those areas as separate populations for the second group of scenarios. The second group of nine scenarios used the population from south of the Yucatán Peninsula as the most recent common ancestor (MRCA) and tested whether Mayan Cichlids in Florida were introduced from Mexico, Guatemala, or Belize, or whether there were multiple introductions from those regions.

For both sets of scenario analyses in DIYABC, we used broadly defined priors as no prior values were known for the parameters (Table 1). We used the Generalized Stepwise Mutation Model [86] with a uniform prior distribution for the mean mutation rate (1E^4^ – 1E^3^). The ‘one sample summary statistics’ used for each population were the mean number of alleles, the mean genetic diversity, mean size variance and, mean Garza-Williamson's M. The ‘two sample summary statistics’ used were compared between population pairs, and included Fst, mean index of classification (the mean individual assignment likelihood of individuals collected in one population and assigned to another population), and (δμ)^2^ genetic distance [87]. For each scenario, 1,000,000 simulated datasets were created. Prior-scenario combinations were evaluated using Principal Components Analysis (PCA) as implemented by the software. Posterior probabilities of scenarios were compared with logistic regression using 1% of the closest simulated datasets, as implemented by DIYABC v. 2.0. Estimations of parameters were also computed and performance of parameter estimates was evaluated by assessing confidence and bias as implemented by the software.

**Table 1:** Prior distribution of parameters used in ABC analyses.

Parameter	Interpretation	Distribution	Minimum	Maximum
N	Effective population size	Uniform	10	100000
Nf	Number of founders for each population	Uniform	2	10000
t1, t2, t3, t4, t5	Time of events in generations	Log-uniform	1	10000
db	Duration of bottleneck in generations	Log-uniform	1	10000
r	Admixture rate	Uniform	0.001	0.999

The time of events in generations are labelled backwards in time and the conditions were as follows: t1<t2<t3<t4<t5.

## Results

### Mitochondrial cytochrome b

**Figure 2:**
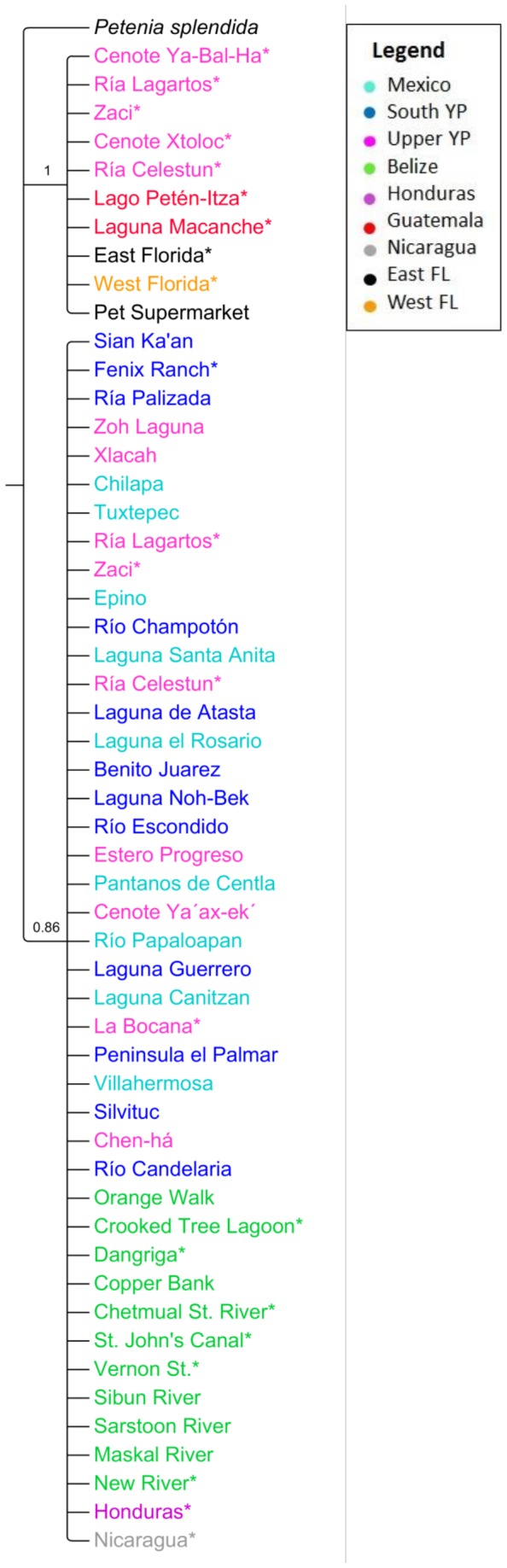
Consensus tree generated by Bayesian phylogenetic analysis using the sister species, *Peténia splendida*, as an outgroup. Clade credibility for branches are shown. Samples that exhibited the same haplotype from East and West Florida, Honduras and Nicaragua were each collapsed into a single branch for clarity. Branches are color-coded by region. * denotes sites where specimens were also analyzed at microsatellite loci.

**Figure 3:**
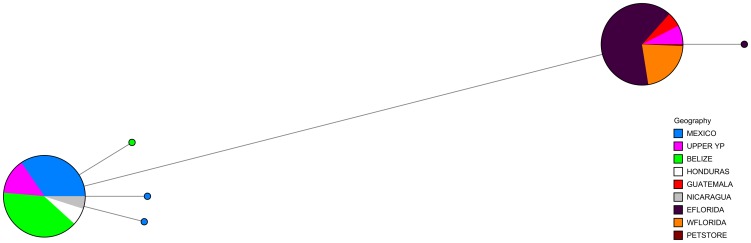
Haplotype network of cytochrome b in Mexico, Central America and Florida. Circles represent different haplotypes; sizes of partitions within circles are proportional to the number of specimens per haplotype. Colors correspond to localities as indicated. Line lengths signify the number of bases separating each haplotype; the short lines symbolize one base and the long line denotes six bases.

Six haplotypes were recovered from sequencing cytochrome b for 47 individuals; the remaining 623 specimens were screened for the CA and Fl haplotypes. The CA and Fl haplotypes differed by six bases within cytochrome b (Genbank accession numbers KM079191 and KM079192). The phylogenetic tree of cytochrome b haplotypes displayed two distinct clades. One clade contained only individuals from the native range, while the second clade contained all the sampled individuals from Florida, some of the individuals from five Mexican sites (Xtoloc, Ya Bal Ha, Zaci, Ria Celestun and Ria Lagartos) and all sampled individuals from two sites in Guatemala (Lago Petén Itza and Laguna Macanche) (Figure 2). Network analyses indicated that the CA haplotype was shared among individuals from Mexico, Belize, Honduras, and Nicaragua while the Fl haplotype was shared among specimens from the eastern and western coasts of Florida, Guatemala and some individuals from Mexico (Figure 3). All but one individual in Florida displayed the same haplotype as the Guatemalan fish; the lone Florida outlier differed from the Fl haplotype by a single base.

### Nuclear microsatellite loci

**Figure 4:**
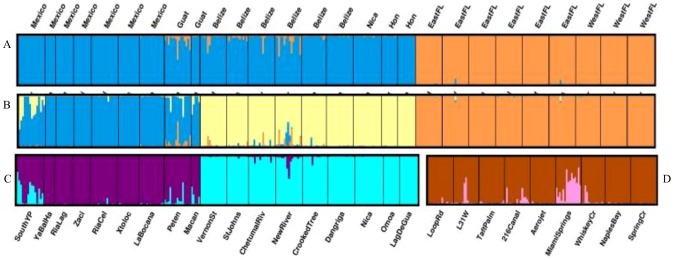
Box plots showing STRUCTURE analysis of Mexico, Central America and Florida for K = 2 (A) and K = 3 (B). Box plots of cluster analysis of sites within Central America for K = 2 (C) and within Florida for K = 2 (D).

Seventeen loci were analyzed for 356 specimens from 29 sites in Florida, the upper Yucatán Peninsula and south of the Yucatán Peninsula in Mexico, Belize, Honduras, Nicaragua and the Petén region of Guatemala. The Belize population exhibited the highest number of effective alleles (6.56) while Florida had the lowest (2.42) (Table 2). Observed and expected heterozygosities were highest in Belize; expected heterozygosity was lowest in Florida and observed heterozygosity was lowest in the upper Yucatán Peninsula (Table 2). Florida specimens exhibited 142 alleles, 42 of which were found in specimens from both Belize and Guatemala, 45 from Belize alone, 11 from Guatemala alone, 11 from sites in Mexico, and 33 were private alleles. The Stepwise Mutation Model did not yield significant levels of heterozygosity excess for Florida sites (Wilcoxon signed-rank one-tail test: p = 1). Structure analysis using the Evanno method [77] indicated that the uppermost levels of differentiation in population structure were for K = 2 (ΔK = 1395.23) and K = 3 (ΔK = 272.83; Figure S1). We presented results for both K values because they were both biologically important and reflected regional hydrology (Figure 4). The uppermost level of differentiation divided all of the samples into two possible populations, the first contained individuals from Florida and the second contained individuals from Mexico and Central America (Figure 4A). When the number of possible populations was three, individuals from Florida remained within a single cluster while individuals from Belize, Honduras and Nicaragua formed a second cluster and individuals from Mexico and Guatemala formed a third grouping (Figure 4B).

**Table 2:** Summary statistics calculated for microsatellite markers.

Region	Location	# of samples	# of different alleles	# of effective alleles	H^o^	H^e^	% of polymorphic loci	Inbreeding coefficient (FIS)
Mexico	South of YP	15	6	4	0.54	0.65	100%	0.13
Mexico	Upper YP	67	11	5	0.33	0.61	94.12%	0.45
CA	Guatemala	20	8	5	0.42	0.73	100%	0.41
CA	Belize	86	16	7	0.57	0.74	100%	0.22
CA	Nicaragua	16	6	4	0.54	0.57	94.12%	0.03
CA	Honduras	18	6	3	0.42	0.58	94.12%	0.29
Florida	Florida	134	8	2	0.35	0.48	100%	0.34

CA denotes Central America; YP denotes the Yucatán Peninsula. # and % represent “number” and “percentage” respectively. H^o^ and H^e^ represent observed and expected heterozygosities.

The two clusters from Florida and Mexico and Central America were analyzed separately by running additional structure analyses. Within the native range grouping, the data were also divided into two clusters (ΔK = 1908.25); the first cluster contained individuals from Mexico and Guatemala while the second contained individuals from Belize, Honduras and Guatemala (Figure 4C). Within Florida, the uppermost level of differentiation divided the data into two clusters (ΔK = 22.74), with individuals from Miami Springs and the L31W canal appearing most similar (Figure 4D). However, examination of clusters for larger K values did not reveal any distinct population structure in Florida.

**Figure 5:**
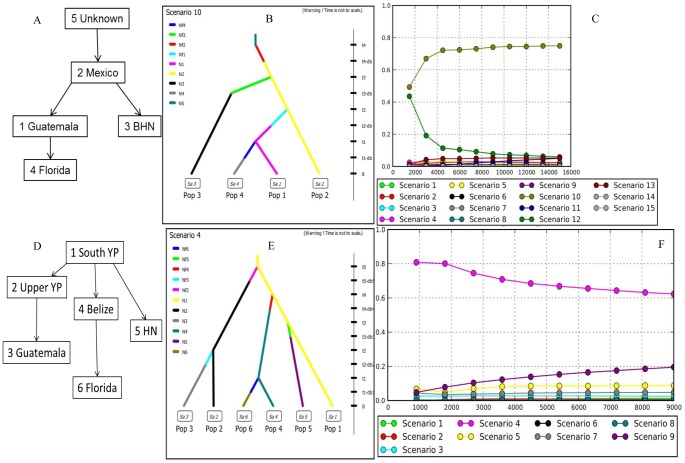
Most supported models and posterior probabilities from groups 1 and 2. Model (A), scenario (B), and logistic regression of posterior probabilities for scenario 10 (C) from group 1, and model (D), scenario (E) and logistic regression of posterior probabilities for scenario 4 from group 2. Population numbers are indicated with the population names in the flow chart. YP refers to Yucatán Peninsula.

Scenario testing analysis of the first group of scenarios showed the highest support for scenario 10, in which fish from an unsampled source were introduced to Mexico, then to both Guatemala and BHN, and then from Guatemala to Florida (Figure 5; Table 3); posterior probability = 0.662, 95% confidence interval (0.617, 0.707). Scenario 10 supported the introduction of Mayan Cichlids from Mexico to Guatemala and BHN (Belize, Honduras and Nicaragua), which was incorporated into the modeled scenarios for the second grouping. Scenario 4 was the most supported from the second grouping of scenarios. In Scenario 4, fish were introduced from southern YP (Yucatán Peninsula) to upper YP, Belize, and the Honduras-Nicaragua group, followed by introductions from Upper YP to Guatemala and from Belize to Florida (Figure 5; Table 3); posterior probability = 0.623, 95% confidence interval (0.514,0.733).

**Table 3:** Median estimates of parameters from group 1, scenario 10 and from group 2, scenario 4.

Parameter	Group 1 Scenario 10	Group 2 Scenario 4
N1	2.02E+03	3.50E+03
N2	8.31E+03	7.70E+04
N3	8.23E+03	5.64E+04
N4	3.09E+03	3.31E+04
N5	2.45E+02	3.54E+04
N6	NA	7.18E+03
Nf2	2.35E+03	2.37E+03
Nf3	4.65E+03	8.90E+03
Nf4	1.64E+03	2.27E+03
Nf5	NA	4.27E+03
Nf6	NA	5.57E+01
t1	2.48E+03	2.69E+03
t2	4.17E+03	6.67E+03
t3	5.30E+03	8.10E+03
t4	5.35E+03	7.55E+03
t5	NA	9.44E+03
db1	4.65E+02	7.37E+03
db2	9.25E+03	6.38E+03
db3	5.35E+03	7.86E+03
db4	7.65E+03	1.17E+03
db5	NA	7.10E+03

For parameters, N = effective population size, Nf = number of founders in each population, t = time of events in generations, and db = duration of bottleneck in generations.

The parameter values correspond to those in Figure 5B and 5E.

NA denotes parameter that were absent in the model.

## Discussion

We observed that the nuclear genetic markers, microsatellites, and the mitochondrial gene, cytochrome b, supported different routes for introduction of Mayan Cichlids into Florida. The nonrandom association of mitochondrial and nuclear alleles, cytonuclear disequilibrium, is strong evidence of introductions of Mayan Cichlids to South Florida through fish from multiple origins [46]–[50]. These data on Mayan Cichlids provides only the second example of which we are aware where cytonuclear disequilibrium provided evidence of multiple introductions in animals [50]. Mayan Cichlids displayed markedly diminished genetic variation in Florida compared to their native range, consistent with a small initial introduction followed by a rapid expansion to their current approximate 70,000 hectare range invaded over 7 to 8 generations. The proposed pattern of introduction from multiple sites, establishment, and expansion can cause cytonuclear disequilibrium [46]–[50]. We also found evidence of movements within Mexico and Central America which is suggestive of human-assisted dispersal.

Phylogenetic analysis and haplotype distribution of cytochrome b indicated an introduction of Mayan Cichlids into Florida from the Petén region of Guatemala or the upper Yucatán Peninsula of Mexico. All but one fish from Florida carried the same cytochrome b haplotype suggesting that either a small number of founders, or low female effective population size carrying the Fl haplotype, were introduced and quickly spread (e.g. [88]). The lone Florida outlier differed from the Fl haplotype by a single base and may represent a post-introduction mutation. Alternatively, the Fl haplotype was fixed in the population after introduction, perhaps through selection or genetic drift acting on a small founder population [89]. The distribution of cytochrome b haplotypes that we found was consistent with research by Razo-Mendivil et al. [90], who sequenced cytochrome b for Mayan Cichlids throughout southern Mexico and Central America and found high genetic structuring corresponding with two highly divergent groups. Unlike their study, we used restriction endonuclease enzyme digestion in lieu of sequencing cytochrome b and thus found fewer cytochrome b haploytpes within Mexico and Central America than their study. However, their most common haplotypes, Cu1 and Cu12, reflected the distributions of CA and Fl haplotypes we observed within Mexico and Central America, confirming the efficacy of our screening methods for phylogenetically useful cytochrome b haplotypes.

The first group of scenarios we tested using ABC supported a pathway whereby Mayan Cichlids were introduced from an unsampled source to Mexico, then to both Guatemala and the cluster of Belize-Honduras-Nicaragua, and then from Guatemala to Florida. Cytochrome b results also supported Guatemala as the introduction source of Mayan Cichlids in Florida because they shared the Fl haplotype. We grouped Belize with Honduras and Nicaragua for the first group of scenarios because of their genetic similarity indicated by the cluster analysis. However, because Belize is within the Usumacinta drainage, unlike Honduras and Nicaragua, and because there was some genetic similarity of individuals between Florida and Belize, we grouped Belize separately for the second set of scenario testing. We investigated whether the ‘unsampled population’ indicated by the most supported scenario from group 1 was representative of a population near the Ria Grijalva basin where the sister species of Mayan Cichlids (*Peténia splendida*; [91], [92]), and perhaps Mayan Cichlids themselves, arose [93]. Thus, we used samples from south of the Yucatán Peninsula as the most recent common ancestral population for the second group of scenarios to improve model fit. Both of the most highly supported scenarios corroborated an introduction from Mexico to Guatemala suggesting that the Fl haplotype spread from Upper Yucatán Peninsula to Guatemala, which was a likely introduction source for Florida (group 1, scenario 10). The most supported scenario from the second group and shared alleles indicated an introduction to Florida from Belize; however, a Belizean introduction is not supported by cytochrome b data because we failed to find the Fl haplotype at any Belize sites.

Our results showed that the Florida population contained a mitochondrial allele from Guatemala and a nuclear lineage most similar to Belize resulting in a form of cytonuclear disequilibrum that is expected when small founding populations that are genetically differentiated at nuclear and mitochondrial loci are admixed [47]–[50]. There was also some genetic similarity in microsatellites between fish from Florida and Guatemala, which is expected if Guatemala was also an introduction source. We were not able to test for cytonuclear disequilibrium within Florida populations using standard methods [49], [94] because we identified only one effective haplotype within Florida (the only other haplotype we found in Florida was in a single individual). We propose that an introduction from Petén occurred, as a result of the aquarium trade [95], [96], where all the females were fixed for the Fl cytochrome b haplotype followed by an introduction from Belize. Cichlid hobbyists and aquarists imported many neotropical cichlid species into the United States starting in the 1970s [96]. The founding population from Belize likely contained mostly males, though we cannot rule out mutation and subsequent selection for the Fl haplotype after introduction resulting in an introduced population that is genetically similar to two distinct populations. Another possibility is that the Fl haplotype was present in the Belize population, but at such low frequencies that we could not identify it within Belize specimens. The breeding of Mayan Cichlids by aquarists and cichlid hobbyists prior to its release in Florida may have facilitated the hybridization of Mayan Cichlids from Guatemala and Belize or the nonrandom mating of females from Guatemala with males from Belize, which may have yielded the cytonuclear disequilibrium we observed.

Based on microsatellite data, Mayan Cichlids within Florida formed two clusters that were not very distinct, indicating low levels of population differentiation among sites in Florida. The relatively high inbreeding coefficient and the low genetic diversity within Florida supports the hypothesis of introduction of a small number of individuals that subsequently spread throughout southern and central Florida at an approximate rate of 2,300 hectares per year (total range of approximately 70,000 hectares) [9]. The relatively large number of private microsatellite alleles within Florida is also an expected result of small introductions and subsequent population expansion if the introduced individuals carried alleles that are currently rare within the native range – and were therefore unsampled in this study – and frequency of those alleles increased in the Florida population as a result of a bottleneck. We used the test for heterozygosity excess to determine the occurrence of a bottleneck because it was more robust to assumptions about mutation models than other bottleneck testing methods [97]. Although our test for a bottleneck in Florida populations did not yield significant results, this does not preclude the occurrence of a historic bottleneck. As effective population size increases after a bottleneck occurs, statistical power to detect the bottleneck decreases even with large sample sizes [97]–[99]. Therefore, if Mayan Cichlids suffered a bottleneck and a subsequent rapid population expansion, the populations would rapidly obtain mutation-drift equilibrium making heterozygosity excess difficult to detect.

### Cytochrome b within Central America

The Fl haplotype was found in all fish from Lago Petén, Laguna Macanche, Cenote Ya-Bal-Ha, and Cenote Xtoloc, and some fish from Ría Lagartos, Cenote Zaci, and Ría Celestun. Although these areas are all part of the Yucatán Division of the Usumacinta Drainage [84], Cenote Ya-Bal-Ha, Cenote Xtoloc, Cenote Zaci, and Ría Celestun are all located in the upper Yucatán Peninsula, which has no major drainages that connect them to the rest of the Usumacinta basin [82], [88] where Mayan Cichlids are believed to have arisen [84], [85], [100], [101]. Dispersal between the Petén region of Guatemala and Upper Yucatán through freshwater channels is possible; a similar pattern was also found for *Gambusia yucatana* where individuals from northern Yucatán Peninsula and Petén were morphometrically more similar than with nearby sites [102]. However, we did not observe the Fl haplotype at any sampling location between Petén and the Upper Yucatán as expected with dispersal. Mayan Cichlids are tolerant of salt water [53], [59], [103], [104] and could have arrived via marine corridors along the coast or during sea level changes during the Pleistocene and early Holocene [90], [101] although the hypothesis of strict marine dispersal by Cichlids is disputed [105]–[108]. It is also possible that Mayan Cichlids were transported between the Upper Yuctán and Guatemala by humans since they have been purposely introduced to many water bodies in Mexico for mosquito control and as a food source [53], [54], [93], [109]–[111]. The sites where the Fl cytochrome b haplotype were found are also near to Maya sites [112]–[114]. Pre-Columbian peoples cultivated freshwater snails as a food source [115], developed artificial fisheries [116], and stocked their reservoirs with fish [117]. As they do today, the Maya would have used this species for food and may have introduced them along their trade routes to water bodies from which they were absent.

### Conclusion

Mayan Cichlids have become established in southern Florida; they have spread and impacted their introduced environment, representing a case of a successful invader that resulted from multiple introductions. Unlike other studies, the introductions from distinct sources did not increase overall genetic diversity compared to the native range. Instead, it resulted in a genetic bottleneck which decreased overall genetic diversity and produced novel combinations of mitochondrial haplotypes and nuclear alleles. Introduction was followed by rapid population growth and dispersal throughout south Florida. This admixture between distinct Belize and Guatemala lineages, probably accomplished while in cultivation in ornamental fish farms, could have improved fitness and facilitated establishment and spread in Florida.

## Supporting Information

Figure S1 **Rate ofchange of the likelihood distribution (mean± SD) from STRUCTURE analysis.** Calculated as L′(K) = L(K)−L(K−1) (see [76]). The highest values are the most supported values of K.
(TIF)

Table S1 **Location and number of Mayan cichlid samples collected at each site.**
(DOCX)

Table S2 **Scenarios 1–15 for group 1 and scenarios 1–9 for group 2 in DIYABC analyses.** Scenarios show the hypothesized movement pathways for Mayan Cichlids (indicated by downward arrows). For all models, time (t) increases upward.
(DOCX)
