# Supplementary figures and images for: Genetic Evidence for Multiple Sources of the Non-Native Fish Cichlasoma urophthalmus (Günther; Mayan Cichlids) in Southern Florida
Source: PLoS One. 2014 Sep 3;9(9):e104173. doi: 10.1371/journal.pone.0104173 (PMC4153574; doi:10.1371/journal.pone.0104173)

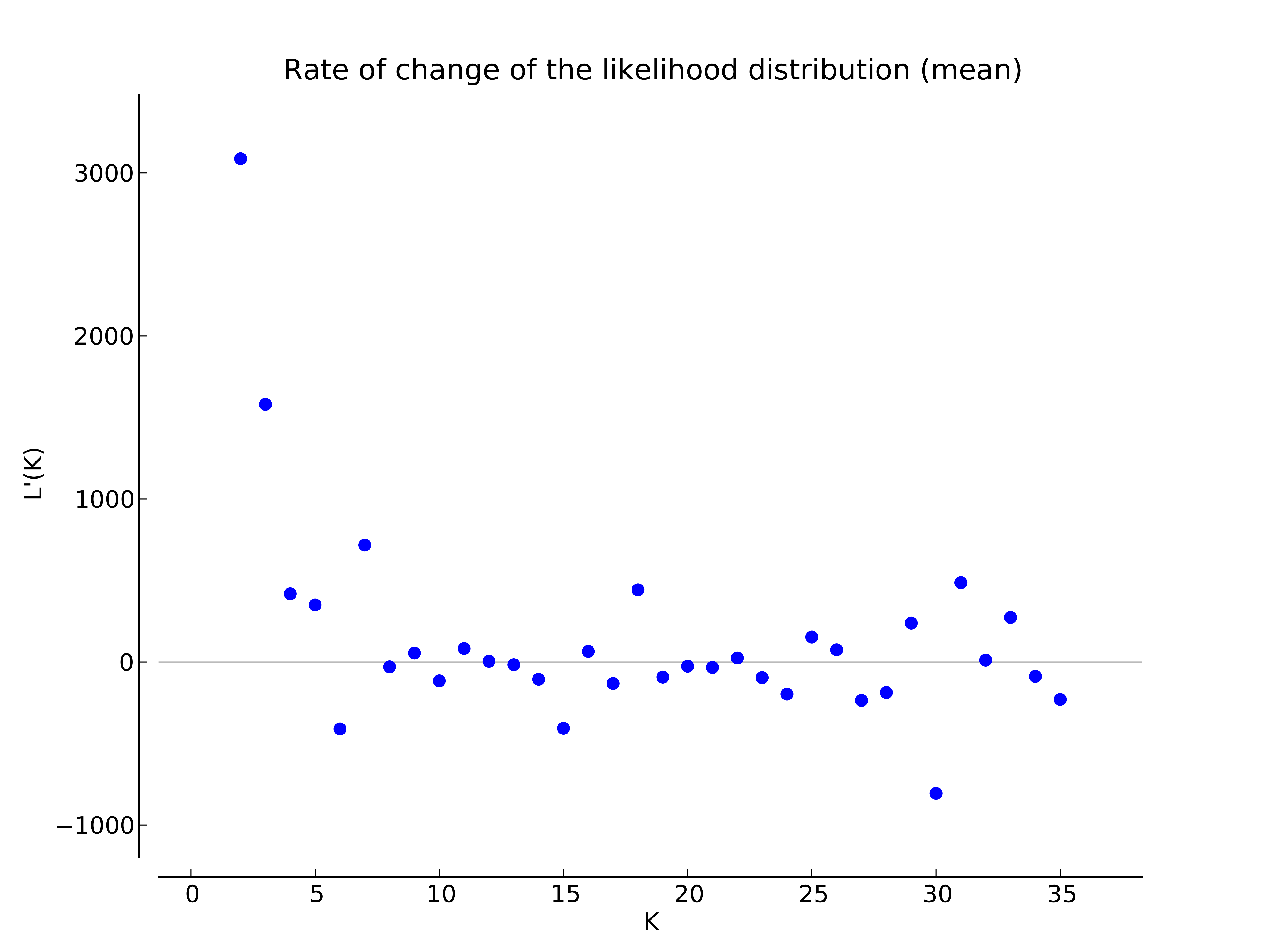

Supplement: Figure S1 — Rate ofchange of the likelihood distribution (mean± SD) from STRUCTURE analysis. Calculated as L′(K) = L(K)−L(K−1) (see [76]). The highest values are the most supported values of K. (TIF) [file pone.0104173.s001.tif]
