# Supplementary material for: Genetic Evidence for Multiple Sources of the Non-Native Fish Cichlasoma urophthalmus (Günther; Mayan Cichlids) in Southern Florida
Source: PLoS One. 2014 Sep 3;9(9):e104173. doi: 10.1371/journal.pone.0104173 (PMC4153574; doi:10.1371/journal.pone.0104173)
Supplement: Table S1 — Location and number of Mayan cichlid samples collected at each site. (DOCX) [file pone.0104173.s002.docx]

**Table S1. Location and number of Mayan cichlid samples collected at each site.**

Coordinates are presented as latitude (Lat.) and longitude (Long.) in decimal degrees. * denotes the sites where specimens were also analyzed at microsatellite loci.

| Site # | Geographical Location | Region | Collection Site/Population | Lat. | Long. | # of samples |
| --- | --- | --- | --- | --- | --- | --- |
| 1 | Mexico | Campeche | Laguna de Terminós* | 18.7111 | -91.634 | 19 |
| 2 |  | Campeche | Ría Palizada | 18.251 | -92.097 | 1 |
| 3 |  | Campeche | Río Champotón | 19.2805 | -90.624 | 9 |
| 4 |  | Campeche | Río Candelaria at Zaragoza | 18.3874 | -91.308 | 4 |
| 5 |  | Campeche | Laguna de Atasta | 18.6192 | -92.102 | 3 |
| 6 |  | Campeche | Peninsula el Palmar | 18.9252 | -91.333 | 9 |
| 7 |  | Campeche | Silvituc | 18.6233 | -90.285 | 1 |
| 8 |  | Oaxaca | Tuxtepec | 18.0792 | -96.12 | 3 |
| 9 |  | Quintana Roo | Sian Ka'an | 19.1988 | -87.888 | 4 |
| 10 |  | Quintana Roo | Benito Juarez | 18.8116 | -88.29 | 2 |
| 11 |  | Quintana Roo | Laguna Noh-Bek | 19.1423 | -88.166 | 4 |
| 12 |  | Quintana Roo | Río Escondido at Ucum | 18.4993 | -88.529 | 4 |
| 13 |  | Quintana Roo | Laguna Guerrero | 18.7031 | -88.252 | 10 |
| 14 |  | Tabasco | Chilapa | 18.1951 | -92.666 | 6 |
| 15 |  | Tabasco | Epino | 18.2487 | -92.837 | 8 |
| 16 |  | Tabasco | Laguna Santa Anita | 18.3836 | -92.902 | 3 |
| 17 |  | Tabasco | Pantanos de Centla | 18.4719 | -92.654 | 5 |
| 18 |  | Tabasco | Laguna El Rosario | 17.8968 | -93.89 | 4 |
| 19 |  | Tabasco | Laguna Canitzan | 17.5847 | -91.389 | 10 |
| 20 |  | Tabasco | Villahermosa | 18.0511 | -92.93 | 1 |
| 21 |  | Upper YP | Ría Celestun* | 20.75 | -90.35 | 7 |
| 22 |  | Upper YP | Zoh Laguna | 20.8399 | -86.898 | 1 |
| 23 |  | Upper YP | Xlacah | 21.0783 | -89.599 | 4 |
| 24 |  | Upper YP | Cenote Ya-Bal-Ha* | 21.5941 | -88.077 | 6 |
| 25 |  | Upper YP | Ría Lagartos* | 21.5984 | -88.157 | 6 |
| 26 |  | Upper YP | Cenote Zaci* | 20.6917 | -88.197 | 10 |
| 27 |  | Upper YP | Cenote Xtoloc* | 20.6801 | -88.569 | 12 |
| 28 |  | Upper YP | Cenote Chen-há | 20.6895 | -89.876 | 4 |
| 29 |  | Upper YP | Estero Progreso | 21.2763 | -89.67 | 6 |
| 30 |  | Upper YP | Cenote Ya´ax-ek´ | 20.6209 | -88.404 | 4 |
| 31 |  | Upper YP | La Bocana* | 21.1933 | -89.953 | 14 |
| 32 |  | Veracruz | Río Papaloapan | 18.6099 | -95.658 | 10 |
| 33 | Belize | Belize | Crooked Tree Lagoon* | 17.7745 | -88.525 | 16 |
| 34 |  | Belize | Maskal River | 17.8806 | -88.313 | 6 |
| 35 |  | Belize City | Chetumal River* | 17.5085 | -88.221 | 15 |
| 36 |  | Belize City | St. John's College Canal* | 17.5116 | -88.199 | 13 |
| 37 |  | Belize City | Vernon Street River* | 17.4976 | -88.196 | 26 |
| 38 |  | Corozal | Copper Bank | 18.3287 | -88.346 | 6 |
| 39 |  | Hattieville | Sibun River | 17.457 | -88.392 | 8 |
| 40 |  | Orange Walk | Orange Walk | 17.7604 | -88.865 | 8 |
| 41 |  | Orange Walk | New River* | 17.5573 | -88.532 | 17 |
| 42 |  | Stann Creek | Dangriga* | 16.9681 | -88.226 | 13 |
| 43 |  | Toledo | Sarstoon River | 15.9564 | -89.003 | 8 |
| 44 | Honduras | Cortés | Omoa* | 15.77 | -87.004 | 8 |
| 45 |  | Colón | Laguna de Guaymoreto* | 15.9951 | -85.905 | 10 |
| 46 | Guatemala | Petén | Lago Petén-Itza* | 16.9948 | -89.822 | 14 |
| 47 |  | Petén | Laguna Macanche* | 16.9667 | -89.633 | 5 |
| 48 | Nicaragua | RAAN | Laguna de Wouhnta* | 13.5991 | -83.527 | 8 |
| 49 |  | RAAN | Puerto Cabezas* | 14.0521 | -83.399 | 5 |
| 50 |  | RAAN | Laguna de Karata* | 13.9264 | -83.501 | 3 |
| 51 | East Florida | Miami Urban Canal | Miami Springs* | 25.822320 | -80.289 | 27 |
| 52 |  | Miami Urban Canal | 216 Canal* | 25.7684 | -80.314 | 19 |
| 53 |  | Miami Urban Canal | Airport Lakes | 25.7862 | -80.271 | 9 |
| 54 |  | Miami Urban Canal | 57th Avenue | 25.7385 | -80.286 | 6 |
| 55 |  | Tamiami Trail | Loop Road* | 25.7598 | -80.766 | 21 |
| 56 |  | Tamiami Trail | Water Conservation Area 3A | 25.7626 | -80.689 | 13 |
| 57 |  | Tamiami Trail | Everglades Gun Range canal | 25.7618 | -80.477 | 8 |
| 58 |  | North of Tamiami Trail | Krome Avenue | 25.9034 | -80.447 | 4 |
| 59 |  | I-75 | Marker 50 | 26.1533 | -81.348 | 6 |
| 60 |  | North Miami | Pet Store |  |  | 2 |
| 61 |  | Broward Urban Canal | Taft Palm Ave* | 26.022340 | -80.28 | 26 |
| 62 |  | Palm Beach Urban Canal | 441 W | 26.272 | -80.202 | 6 |
| 63 |  | ENP | L31W* | 25.4546 | -80.563 | 32 |
| 64 |  | ENP | Aerojet Canal* | 25.3711 | -80.539 | 18 |
| 65 |  | ENP | C-111 Canal | 25.3432 | -80.576 | 8 |
| 66 | West Florida | Corkscrew Swamp | Corkscrew | 26.4428 | -81.808 | 10 |
| 67 |  | Charlotte Harbor | Punta Gorda Ditch | 26.9597 | -81.95 | 1 |
| 68 |  | Estero Bay | Spring Creek* | 26.3886 | -81.853 | 15 |
| 69 |  | Fort Myers | Montego Bay Condominiums | 26.6936 | -81.725 | 1 |
| 70 |  | Fort Myers | Henry Creek | 26.5177 | -81.875 | 11 |
| 71 |  | Fort Myers | Coral Waters | 26.5705 | -81.836 | 10 |
| 72 |  | Fort Myers | Whiskey Creek* | 26.5844 | -81.879 | 19 |
| 73 |  | Naples | Naples Bay* | 26.1664 | -81.787 | 15 |

YP represents Yucatán Peninsula; RAAN represents Región Autonoma Atlántico Norte. * denotes samples that were included in microsatellite analysis.
