# Supplementary material for: Genetic Evidence for Multiple Sources of the Non-Native Fish Cichlasoma urophthalmus (Günther; Mayan Cichlids) in Southern Florida
Source: PLoS One. 2014 Sep 3;9(9):e104173. doi: 10.1371/journal.pone.0104173 (PMC4153574; doi:10.1371/journal.pone.0104173)
Supplement: Table S2 — Scenarios 1–15 for group 1 and scenarios 1–9 for group 2 in DIYABC analyses. Scenarios show the hypothesized movement pathways for Mayan Cichlids (indicated by downward arrows). For all models, time (t) increases upward. (DOCX) [file pone.0104173.s003.docx]

**Table S2. Scenarios 1-15 for group 1 and scenarios 1-9 for group 2 in DIYABC analyses** Scenarios show the hypothesized movement pathways for Mayan Cichlids (indicated by downward arrows). For all models, time (t) increases upward.

| Scenario Number | Group 1 | | Group 2 | |  |
| --- | --- | --- | --- | --- | --- |
| 1 | 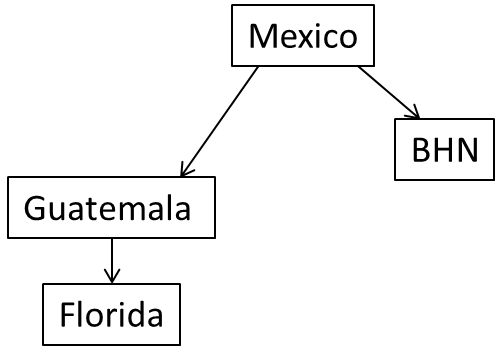 | | 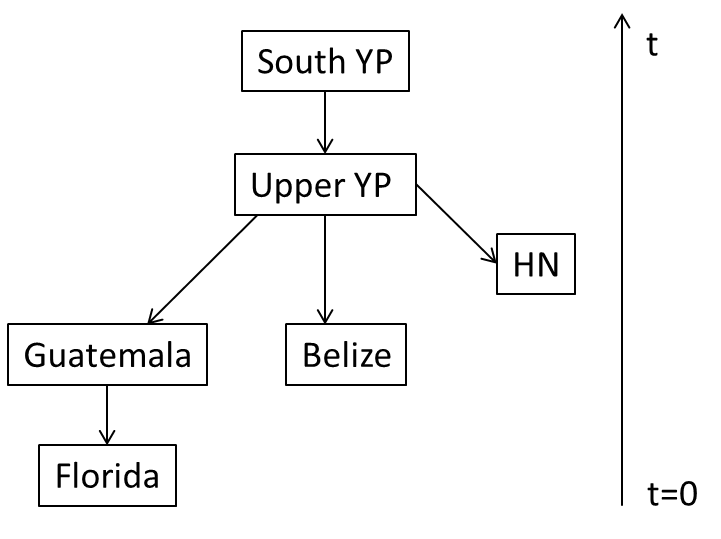 | |  |
| 2 | 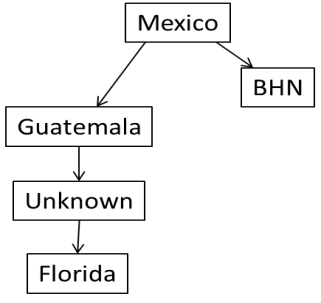 | | 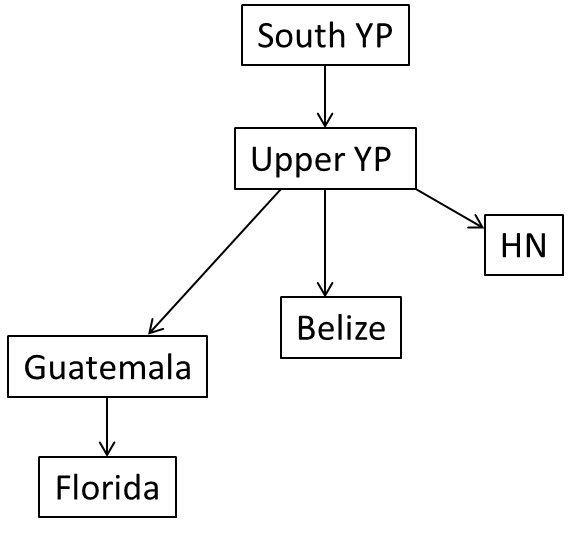 | |  |
| 3 | 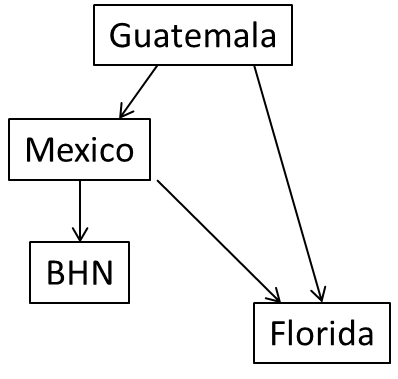 | | 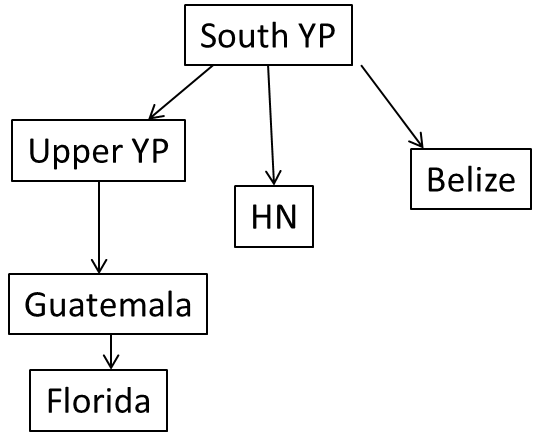 | |  |
| 4 | 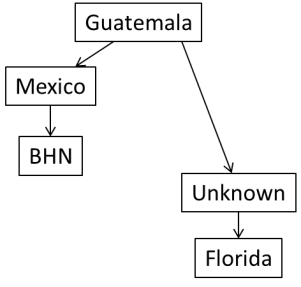 | | 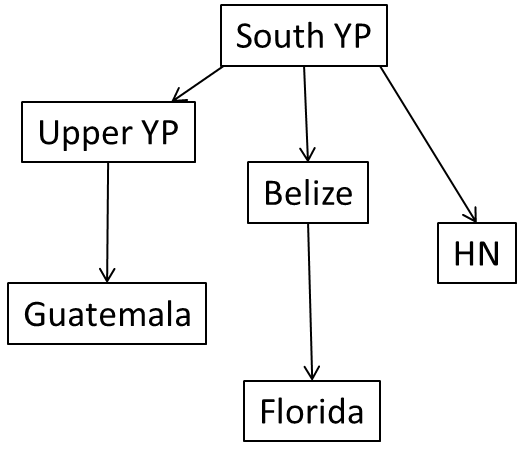 | |  |
| 5 | 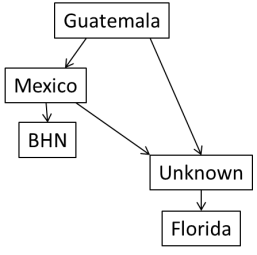 | | 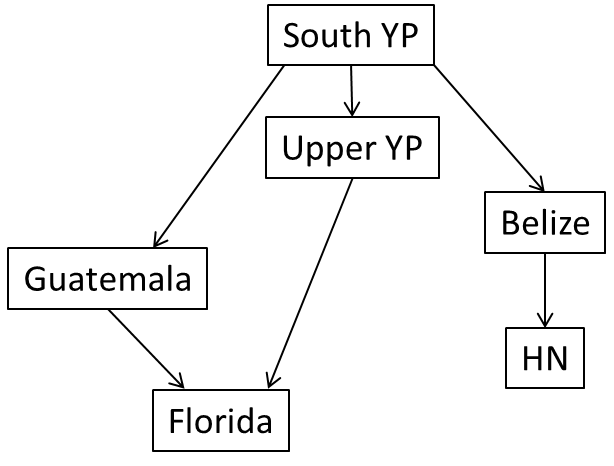 | |  |
| 6 | 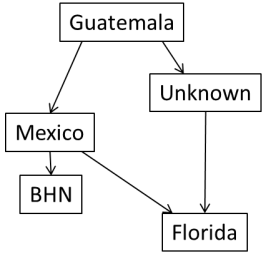 | | 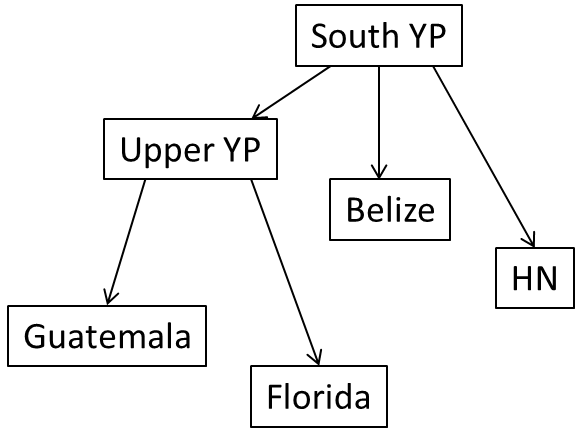 | |  |
| 7 | 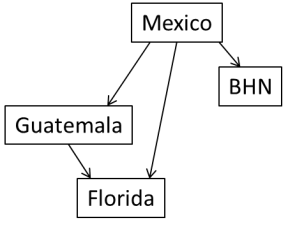 | | 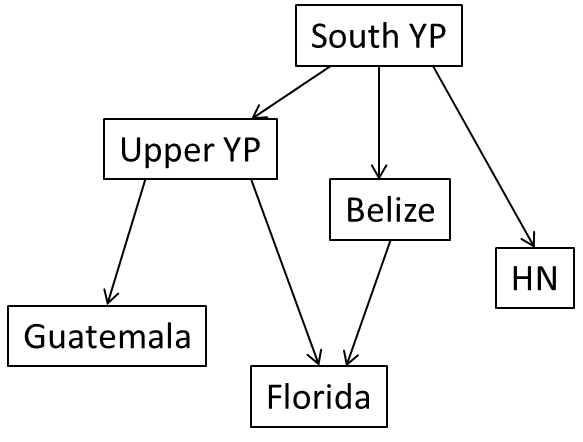 | |  |
| 8 | | 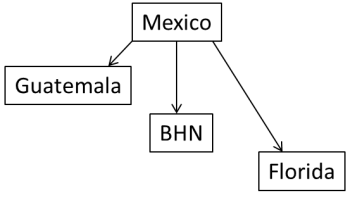 | | 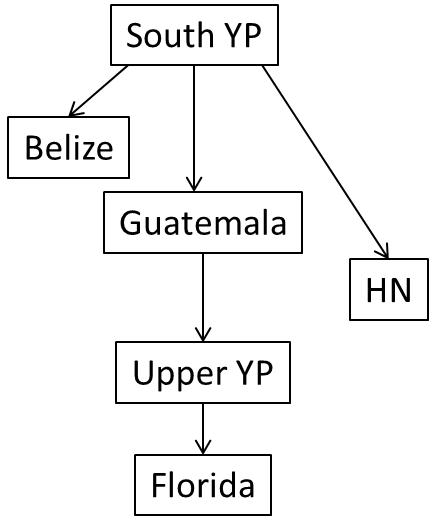 | |
| 9 | | 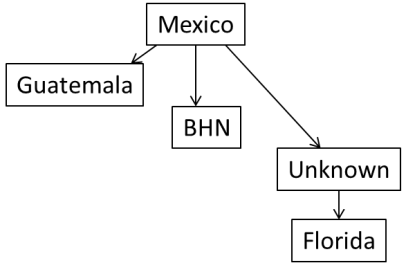 | | 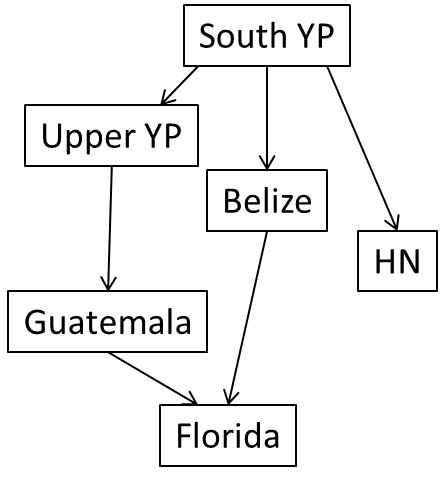 | |
| 10 | | 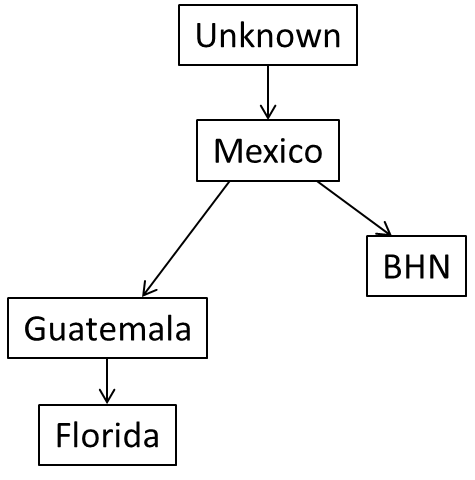 | |  |  |
| 11 | | 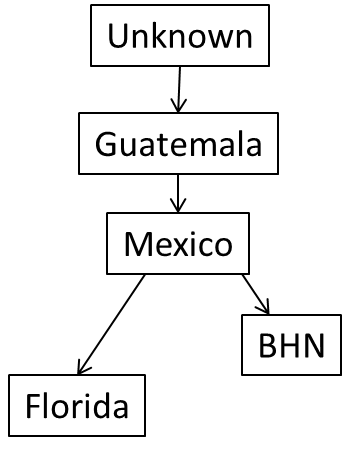 | |  |  |

| 12 | 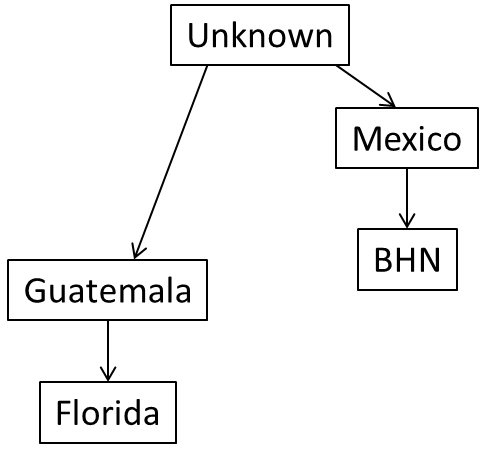 |
| --- | --- |
| 13 | 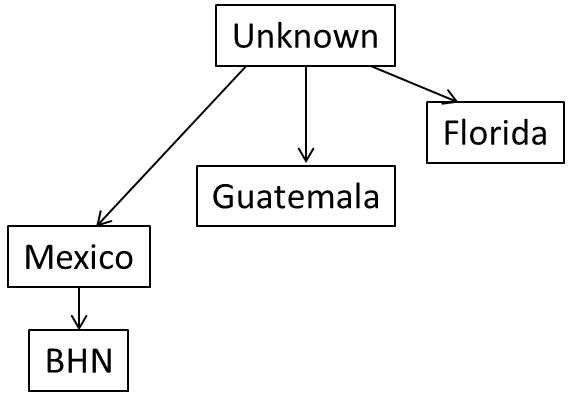 |
| 14 | 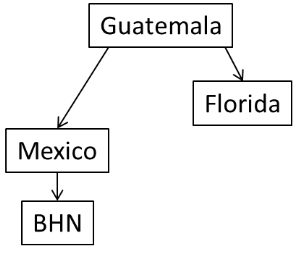 |
| 15 | 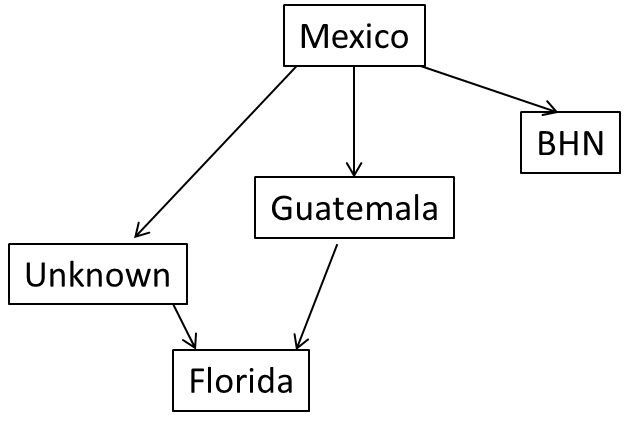 |
